# *CYP19A1 TC/CC* Polymorphism, along with Deletion of *GSTM1* and *GSTT1* Genes, Strongly Influences Female Infertility Risk

**DOI:** 10.3390/antiox12040940

**Published:** 2023-04-16

**Authors:** Maria Manuel Casteleiro Alves, Micaela Almeida, António Hélio Oliani, Luiza Breitenfeld, Ana Cristina Ramalhinho

**Affiliations:** 1 Health Sciences Research Centre (CICS), Faculty of Health Sciences, University of Beira Interior (UBI), 6201-506 Covilhã, Portugal; mariamanuelcasteleiro@gmail.com (M.M.C.A.); micaelacpalmeida@gmail.com (M.A.); aholiani@gmail.com (A.H.O.); luiza@fcsaude.ubi.pt (L.B.); 2 Assisted Reproduction Laboratory of Academic Hospital of Cova da Beira (CHUCB), 6200-251 Covilhã, Portugal; 3 Department of Obstetrics and Gynecology, São José do Rio Preto School of Medicine (FAMERP), São José do Rio Preto 15090-000, Brazil

**Keywords:** infertility, polymorphisms, *CYP19A1*, *GSTM1*, *GSTT1*, oxidative stress

## Abstract

Oxidative stress has a fundamental role in the pathophysiology of various conditions, like infertility. This case-control study was performed to assess the potential role of *CYP19A1*, *GSTM1*, and *GSTT1* in modifying individual predisposition to female infertility. Genotyping of 201 women with established infertility and 161 fertile female controls was performed, and statistical associations were analyzed. For carriers of *GSTM1* null genotype along with *CYP19A1 C* allele, there is a significant association with female infertility risk (OR 7.023; 95% CI (3.627–13.601; *p* < 0.001), and, also for carriers of *GSTT1* null genotype along with the *CYP19A1 TC/CC* genotype (OR 24.150; 95% CI (11.148–52.317; *p* < 0.001). A positive association with female infertility risk for carriers of the *C* allele in *CYP19A1* and null genotypes in *GTSM1* (OR 11.979; 95% CI (4.570–31.400; *p* < 0.001) or *GSTT1* (OR 13.169; 95% CI (4.518–38.380; *p* < 0.001) was found. When both *GSTs* are deleted, the risk of developing female infertility is significant, independently of the *CYP19A1* genotype; when all the presumed high-risk genotypes are present, we found a significant association with female infertility risk (OR 47,914; 95% CI (14,051–163,393; *p* < 0.001).

## 1. Introduction

Infertility is one of the most important medical conditions around the globe. About 80 million people suffer from infertility [1,2,3,4]. About one in every ten couples develops primary or secondary infertility in the world. Centered on medical, sexual, and reproductive history, age, physical exam results, and diagnostic tests, fertility interventions can be initiated in less than a year [5,6]. There is growing evidence that the interactions between genetic and environmental factors may be implicated in the pathogenesis of infertility [7,8,9]. In addition, the multifactorial nature of this disease means that its incidence may differ between different ethnic groups. Infertility may have a female cause or a male cause or be related to factors from both elements of the couple; despite the origin of the cause, infertility is considered to be a disease of a couple and not a disease of an individual itself [6]. Oligozoospermia, asthenozoospermia, azoospermia, teratozoospermia, and varicocele are known causes of male-caused infertility [10,11]. Concerning female infertility, it may be caused due to several factors, namely endometriosis, polycystic ovary syndrome (PCOS), and premature ovarian failure (POF). Fallopian tube pathologies can also be responsible for infertility [12]. Up to a third of couples are diagnosed with infertility of unidentified cause [13].

The presence of endometrial tissue outside the uterine cavity is defined as endometriosis. Several studies indicate that endometriosis is a polygenic and multifactorial disease. Lifetime exposure to elevated concentrations of circulating estrogen is an established risk factor for various diseases, including infertility [7,14,15,16]. Some studies report that the high risk of endometriosis may be associated with single nucleotide polymorphisms (SNPs) that are involved in the biosynthesis and metabolism of sex steroids [17,18]. PCOS is a hormonal condition that is present in five to ten percent of women of reproductive age. It is associated with reproductive, metabolic, and psychological dysfunctions. The etiology of PCOS is unknown [19,20,21]; however, studies have shown that genetic factors may predispose certain women to develop this disorder [22]. POF happens when ovarian function comes to an end at or before age 40, with elevated gonadotropins and decreased estrogen levels [23,24]. The cause of POF, in most cases, is idiopathic [25]. Tubal factor is another cause of female infertility. Various pathological conditions affecting the fallopian tubes can interfere with the normal transport of eggs through the fallopian tubes [26]. Furthermore, studies have shown that fallopian tubes are dependent on estrogen for morphological and functional integrity [27,28].

Low penetrance genes can be found in several pathways, like metabolization of environmental toxic compounds, metabolism of steroid hormone, and repair of deoxyribonucleic acid (DNA) impairment. *CYP19A1*, in the estrogen biosynthetic pathway, presents interindividual variability, given by different polymorphisms. It is located on chromosome 15q21.1, contains 10 exons and encodes aromatase, the enzyme that catalyzes the final stage of estrogen biosynthesis. *CYP19A1* is a member of the cytochrome P450 mono-oxygenases superfamily that catalyzes numerous reactions in steroidogenesis. The *CYP19A1* gene is expressed in ovaries, placenta, adipose tissue, testes, skin, and sites of the brain, including the hippocampus, amygdala, and hypothalamus [29,30]. Variations of aromatase activity can be correlated to a polymorphism in the *CYP19A1* gene that causes a tryptophan/arginine (Trp/Arg) amino acid substitution at codon 39 of exon 2 (rs2236722), which results in three genotypes *TT*, *TC* and *CC* [31,32]. There is great heterogeneity in the literature about the role of *CYP19A1* gene variants and, consequently, in the difference that these genotypes cause in aromatase activity; there are several polymorphisms in *CYP19A1* that are associated with aromatase deficiency and others related to *CYP19A1* aromatase excess syndrome.

Glutathione S-transferases (*GSTs*) catalyze the conjugation of glutathione (GSH) to various exogenous and endogenous substances containing electrophilic functional groups, such as carcinogens, reactive oxygen species (ROS), and chemotherapeutics. When bound to glutathione, electrophilic compounds become more soluble, allowing for easier and faster elimination. *GST* isozymes are divided into eight classes encoded by the genes *GSTA*, *GSTM*, *GSTK*, *GSTO*, *GSTP*, *GSTS*, *GSTT*, and *GSTZ*, respectively. Furthermore, each class comprises multiple isozymes, each of them encoded by one particular gene [13]. The recognized significance of *GSTs* in the elimination of toxic compounds and in protection against oxidative stress [33] validates more search for relations with the risk of developing different diseases. The *GSTM1* gene is located on chromosome 1p13.3. *GSTT* gene divides into two subunits, *GSTT1* and *GSTT2*, that are located on chromosome 22q11. Polymorphisms at the *GSTM1* and *GSTT1* locus are caused by complete deletions that result in a lack of enzyme activity in individuals with the null genotype [13]. These deletions are likely caused by homologous recombination events. The *GSTM1* and *GSTT1* null genotypes have functional meaning, that is lack of enzymatic activity. Individuals with homozygous deletions in the *GSTM1* and *GSTT1* locus do not display any functional enzymatic activity of the cytosolic enzyme [13]. The balance between ROS levels and antioxidant defenses makes an optimum state for the execution of cellular functions. ROS serve as important second messengers regulating intracellular pathways, and a discrepancy between ROS and antioxidant protection arrangements induces oxidative stress [34]. Several studies have shown that oxidative indicators are expressively augmented in PCOS patients compared to controls, being taken as possible causes of PCOS pathogenesis [19]. Additionally, ROS can disturb several biological functions of the reproductive tract, and high concentrations may cause serious lesions affecting female reproduction [33]. Therefore, GST is thought to play a crucial role in cellular protection from toxic foreign chemicals and oxidative stress.

Taking all the precious information into account, we hypothesized that these polymorphisms might be associated with infertility. To validate this premise, we conducted a case-control study to assess the putative role of *CYP19A1*, *GSTM1,* and *GSTT1* in the modulation of individual predisposition to female infertility.

## 2. Materials and Methods

### 2.1. Study Population

**Figure 1:**
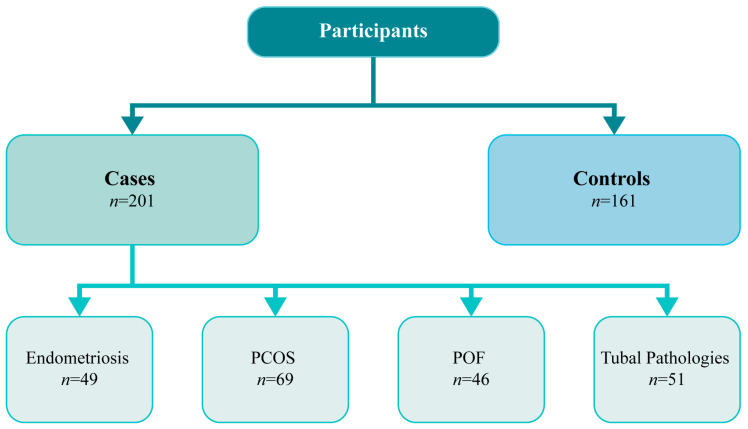
Flowchart diagram of participants of the study.

A total of 201 case samples from women under the age of 39 diagnosed with female infertility were collected., Infertility was defined as the failure to achieve a clinical pregnancy after one year of regular and unprotected sexual activity, diagnosed at Assisted Reproduction Unit from Child and Women Health Department of Academic Hospital Center of Cova da Beira, Covilhã—Portugal. Women were recruited between October 2015 and July 2019. The subjects were mostly Caucasian. We decided to divide the group of cases by the various factors of infertility so that we could also compare the polymorphism of the *CYP19A1*, *GSTM1*, and *GSTT1* genes with these factors individually, resulting in 49 women with endometriosis, 69 women with PCOS, 46 women with POF and 51 women with tubal pathologies (Figure 1). There are women who have more than one associated infertility factor. Male infertility factors were not diagnosed in the group of cases. A control group of 161 fertile women with no gynecological antecedents suitable for infertility and no history of in vitro fertilization (IVF) treatment was selected at obstetrical consultation. Individuals with previous or actual history of osteoporosis, fibroids, breast, endometrial, or other gynecological tumors were excluded.

### 2.2. Ethical Approval

Approval by the Ethical Committee of Academic Hospital Center of Cova da Beira, Covilhã—Portugal was obtained (reference number 47/2015, 15 July 2015). Assignment of informed consent by the participants was performed previously to ingoing the study.

### 2.3. DNA Extraction and Genotyping

Collection of blood was made by venous puncture in ethylenediamine tetraacetic acid (EDTA)-tubes. Extraction of genomic DNA was performed with ReliaPrep^TM^ Blood gDNA Miniprep System Kit (Promega, Fitchburg, WI, USA) according to the manufacturer’s protocol and stored at 4 °C. *CYP19A1* genotyping was accomplished by polymerase chain reaction (PCR) with confronting two-pair primers, adapted from the procedure described by Ramalhinho et al. (2012) and Hirose et al. (2004) [35,36]. The fragments of the *CYP19A1* were amplified with the primers *CYP19A1* forward 1: 5′-ATCTGTACTGTACAGCACC-3′ and reverse 1: 5′-ATGTGCCCTCATAATTCCG-3′, *CYP19A1* forward 2: 5′-GGCCTTTTTCTCTTGGTGT-3′ and reverse 2: 5′-CTCCAAGTCCTCATTTGCT-3′ (Table 1). Briefly, each PCR reaction mixture was carried out in a total volume of 25 μL and contained 10pmol of each primer, 1.5 mM of MgCl_2_, 100 nM of each deoxynucleotide triphosphate (dNTPs), 1 unit of Taq DNA polymerase (Promega, USA), and 100 ng of genomic DNA, using My Cycler Thermal Cycler (Bio-Rad, Munich, Germany). Reaction mixtures were pre-incubated for 10 min at 95 °C. PCR conditions were 1 min at 95 °C, 1 min at 56 °C, and 1 min at 72 °C for 30 cycles. The final extension was at 72 °C for 5 min. The amplified DNA was electrophoresed through 2% agarose gels stained with GreenSafe and run at 120 V for 45 min. Genotypes were distinguished by the presence of a 200-bp band for the T allele, a 264-bp band for the *C* allele, and a 427-bp common band. *GSTM1* and *GSTT1* genotyping was performed using PCR-based methods slightly modified as previously published by our group [37]. The fragments of the *GSTM1/T1* were amplified with the primers *GSTM1* forward: 5′-GAACTCCCTGAAAAGCTAAAG-3′and reverse: 5′GTTGGGCTCAAATATACGGTGG-3′, *GSTT1* forward: 5′TTCCTTACTGGTCCTCACATCTC-3′ and reverse: 5′-TCACCGGATCATGGCCAGCA-3′ and *β-globin* forward: 5′-CAACTTCATCCACGTTCACC-3′ and reverse 5′-GAAGAGCCAAGGACAGGTAC-3′ (Table 2). The presence of wild-type and/or null alleles was analyzed by multiplex PCR together with the co-amplification of a fragment of the *β-globin* gene as a positive control. In brief, each PCR reaction mixture was carried out in a total volume of 25 μL and contained 400 nM of each primer, 1.5 mM MgCl_2_, 100 nM of each deoxynucleotide triphosphate (dNTPs), 1 unit of Taq DNA polymerase (Promega, USA), and 100 ng of genomic DNA (quantified by a spectrophotometric method), using MyCycler Thermal Cycler (Bio-Rad, Munich, Germany). Reaction mixtures were pre-incubated for 5 min at 94 °C (94 °C for 30 s, 57 °C for 30 s, 72 °C for 30 s) × 35 cycles and 72 °C for 5 min. The amplified DNA was electrophoresed through 2% agarose gels stained with GreenSafe, and run at 120 V for 45 min. The fragment size expected was for *GSTM1*: 215 bp; for *GSTT1*: 480 bp, and for *β-globin*: 268 bp.

The methodology applied identified homozygous carriers of *GSTM1* or *GSTT1* deletions. In general, when no PCR products were obtained, individuals were classified as *GSTM1* or *GSTT1* null/null genotypes. The appearance of PCR products in the electrophoresis identifies individuals as *GSTM1* or *GSTT1* “present” in homozygosity or heterozygosity. This method does not distinguish between homozygous wild-type and heterozygous present/null individuals, but it does allow conclusive the identification of null/null genotypes. Results were validated by repetition of genotyping of 10% of the samples by random. Results were consistent with the previously obtained.

### 2.4. Statistical Analysis

The logistic regression method was employed to obtain odds ratios (ORs) and 95% confidence intervals (95% CI) as estimates of relative risk. Chi-squared tests were performed, and *p*-values less than 0.05 were considered statistically significant. Calculations were performed using the computer software Statistical Package for the Social Sciences (SPSS^®^) for Windows (version 24), IBM Corp, Amonk, NY, USA.

## 3. Results

Clinicopathological characteristics of cases (infertile women) and controls (fertile women) are shown in Table 3. The mean age was 34 years (range, 19–39 years) for the cases and 31 years (range, 19–43) for controls. Comparing the age of cases and the age of controls, we found a statistically significant difference (*p* < 0.001). Concerning the number of previous pregnancies, we found that there was a statistically significant association when we compared women with two previous pregnancies and primiparous women (*p* < 0.001), as well as when we compared the number of children of cases with the number of children of controls (*p* = 0.003). These results are compatible with the baseline characteristics of cases and controls. All other baseline characters are similar; that is, the comparative parameters are not significantly different.

Heterozygous and homozygous individuals for the *C* allele were grouped for this analysis. OR, CI, and *p*-values were obtained from the numbers of cases and not for percentages. The distribution of *CYP19A1* codon 39 genotypes in infertile women with endometriosis and in fertile women are shown in Table 4. The frequency of the *CYP19A1 TC/CC* genotype was 40.4% in controls and 75.5% in cases. Significant statistical association of the *TC/CC* genotype combined with endometriosis risk, with reference to the *TT* genotype, was documented (OR 4.554; 95% CI 2.209–9.386; *p* < 0.001).

Additionally, we analysed the distribution of *CYP19A1* codon 39 genotypes in infertile women with PCOS and in fertile women (Table 5). The frequency of *CYP19A1 TC/CC* genotype was 78.3% in cases. In this comparison, we found an increased risk of developing PCOS associated with the *TC/CC* genotype (OR 5.317; 95% CI (2.767–10.215; *p* < 0.001).

The distribution of *CYP19A1* codon 39 genotypes in infertile women with POF and in fertile women are shown are Table 6. The frequency of *CYP19A1 TC/CC* genotype was 69.6% in cases. We observed an increased prevalence of POF with the *TC/CC* genotype (OR 3.376; 95% CI (1.672–6.815; *p* = 0.001).

Lastly, we analysed the distribution of *CYP19A1* codon 39 genotypes in infertile women with tubal pathologies and in fertile women (Table 7). The frequency of *CYP19A1 TC/CC* genotype was 68.6% in cases. We verified an increased prevalence of tubal pathology in carriers of the *TC/CC* genotype (OR 3.231; 95% CI (1.653–6.314; *p* = 0.001).

Considering these results, we analysed the distribution of *CYP19A1* codon 39 genotypes in infertile women and in fertile women, nevertheless the related cause of infertility (*n* = 201). In the comparison of the distribution of *CYP19A1* genotypes in cases and controls (Table 8), we found that the frequency of *CYP19A1 TC/CC* genotype was 68.6% in cases versus 40.4% of the controls; this shows a statistically significant association of *CYP19A1 TC/CC* genotype with infertility, despite of the cause (OR 4.232; 95% (2.710–6.609); *p* < 0.001)

To explore if the pattern of *GSTs* and *CYP19A1* genotypes could be associated with the risk of female infertility, we studied combinations of genotypes. The reference group comprised individuals with all supposed low-risk genotypes, *GSTM1* plus *GSTT1* present and *CYP19A1 TT*. *C* allele carriers in homozygosity or heterozygosity were grouped for this analysis due to the low rate of homozygous *C* allele genotypes and to rise in statistical power. We analysed the two-way combination of *GSTM1* and *CYP19A1* genotypes (Table 9) and found that when *GSTM1* is present, there is a significant association with infertility risk (OR 3.216; 95% CI (1.715–6.031; *p* < 0.001). For *GSTM1* null genotype carriers along with the *CYP19A1 C* allele, there is also a significant association with female infertility risk (OR 7.023; 95% CI (3.627–13.601; *p* < 0.001), but for individuals with simultaneous *CYP19A1 TT* genotype, there is no significant increase in female infertility risk (OR 1.216; 95% CI (0.618–2.392; *p* = 0.346).

About the combined analysis of *GSTT1* and *CYP19A1* genotypes (Table 10), we observed a significant association with female infertility in carriers of the mutated alleles of *CYP19A1* even if *GSTT1* is present (OR 9.143; 95% CI (4.752–17.591; *p* < 0.001), as well as it was also verified a significant association with female infertility with *GSTT1* deletion (OR 17,267; 95% CI (7.366–40.476; *p* < 0.001) and for women with both *CYP19A1* and *GSTT1* mutations (OR 24,150; 95% CI (11.148–52.317; *p* < 0.00).

The combined analysis of *CYP19A1*, *GSTM1*, and *GSTT1* polymorphisms (Table 11) showed an association with female infertility risk for carriers of *GSTM1* and *GSTT1* present genotype (OR 7.108; 95% CI (2.777–18.197; *p* < 0.001). A positive association was also encountered for carriers of the *C* allele in *CYP19A1* along with null genotypes in *GTSM1* (OR 11.979; 95% CI (4.570–31.400; *p* < 0.001) or *GSTT1* (OR 13.169; 95% CI (4.518–38.380; *p* < 0.001), as well as we found an increase in female infertility risk for carriers *CYP19A1 TT* genotype, for individuals with *GSMT1* null genotypes (OR 27.857; 95% CI (7.283–106.551; *p* < 0.001) or *GSTT1* null genotypes (OR 9.471; 95% CI (3.086–29.067; *p* < 0.001). Additionally, when *GSTM1* and *GSTT1* are deleted, there is a significant association with the risk of female infertility; this risk is independent of the *CYP19A1* genotype. The combination of all presumed high-risk genotypes, *CYP19A1 TC/CC*, *GSTM1* deletion, and *GST11* deletion, refers to a statistically significant association with female infertility risk (OR 47.914; 95% CI (14.051–163.393; *p* < 0.001).

## 4. Discussion

In the present study, we have evaluated if women carriers of *CYP19A1* codon 39 Trp/Arg (*T*/*C*) polymorphism (rs2236722) present increased susceptibility to infertility factors. We found a statistically significant difference when comparing the mean age of cases with the mean age of controls. We also found that there is a statistically significant association when comparing women with two previous pregnancies and primiparous women, so as when the number of children of cases with the number of children of controls was compared. Stress levels could explain the higher cfDNA levels in older women, possibly because of the general awareness of higher risks associated with pregnancy at an older age and the known relationship between age and decreased pregnancy success. Relaxation techniques have been shown to be beneficial in reducing plasma cfDNA levels and improving pregnancy outcomes during IVF [38]. Therefore, chronic non-pregnancy stress can lead to increased apoptotic and necrotic events in follicular cells [39].

Our results indicate that carriers of the *TC/CC* genotype in *CYP19A1* appear to be more susceptible to developing endometriosis. Studies have shown that a higher expression of P450 cytochrome in ectopic endometrium increases estrogen levels, thereby activating endothelial cells in the stroma and accelerating the development of endometriosis [40,41]. Estrogen receptors act as transcriptive factors that play a key role in the growth and differentiation of endometrial cells and in the various biological functions in both eutopic and ectopic endometrium [42,43,44]. As far as we know, only one previous study examined the role of this polymorphism in the pathogenesis of endometriosis. This study reported that *CYP19A1* codon 39 Trp/Arg (*T*/*C*) polymorphism (rs2236722) was not significantly associated with the risk of endometriosis [45]. However, it is noteworthy that this study was carried out among Asians, which is a strictly limited population, that can bring confusing effects from interracial differences in genetic backgrounds and environmental factors, such as lifestyle. We did not find any more studies regarding the polymorphism that we studied; however, there are studies on other polymorphisms of the *CYP19A1* gene that corroborate our results. Wang et al. (2012) reported that single nucleotide polymorphisms of the *CYP19A1* (rs700519) gene might modulate the risk of endometriosis. The study shows that homozygous and heterozygous genotypes of rs700519 were at higher risk of developing endometriosis [46]. The study of Vietri et al. (2009) reported a significant prevalence of homozygotes A of *CYP19A1* Val89 polymorphism in women with endometriosis [47]. Two studies indicated an association of rs2899470 and 1531 G > A of the *CYP19A1* gene with endometriosis [48,49]. Therefore, these findings lead us to suggest that this polymorphism may play a role in the increased risk for endometriosis.

We also found that carriers of the *TC/CC* genotype in *CYP19A1* seem to be more susceptible to develop PCOS. Based on our information, just one report examines the role of this polymorphism in the pathogenesis of PCOS. It reported any substantial difference in the frequency of different *CYP19A1* (Trp39Arg) genotypes between PCOS patients and controls [50]. Once again, this study was done in Iran, a population that is different from the Portuguese both in genetic backgrounds and environmental factors, such as living behaviors. The same study reported different polymorphism in the *CYP19* gene (rs2414096) to be associated with the risk of PCOS [51]; also, a study by Wang et al. (2011) reported that the rs700519 polymorphism alters the risk of PCOS [52]. Lazaros et al. (2012) reported that cytochrome P450 aromatase enzyme disturbs estrogen secretion and androgen bioavailability. Thus, aromatase activity can regulate the biosynthesis of estrogens and androgens [53]. The maintenance of an environment dominated by androgen or estrogen is dependent on the activation or inhibition of the aromatase pathway [54,55]. Through ovarian theca cell differentiation, *CYP19A1* polymorphisms lead to an imbalance of androgens and estrogens and can cause a hyperandrogenic phenotype [55,56]. Aromatase activity is decreased in PCOS follicles, leading to abnormal follicle development [57]. Furthermore, variations in *CYP19A1* influence the amount of testosterone available for androgen receptor binding as well as the amount of estrogen available for receptor binding [57]. So, the increase of androgens is a consequence of variation in *CYP19A1* and can be associated with the hyperandrogenic phenotype characteristic of women with PCOS. Considering all these explanations, it is reasonable to speculate that this polymorphism might influence the risk of PCOS.

About POF, our study demonstrated that an increased risk of developing POF appears to be associated with *TC/CC* genotype. Clinically, a woman with PCOS has elevated gonadotropin and low estrogen levels [23,58]. As we mentioned earlier, genetic factors can influence complex diseases through their effects on gene regulation or differential splicing [59,60]. There are several genes involved in the development of POF, including the *CYP19A1* gene [23]. The *CYP19A1* gene codes for an aromatase enzyme that is responsible for converting androgens into estrogens. The polymorphism of this gene can cause aromatase deficiency syndrome, which causes a maturation arrest of follicles. This theory is supported by a study indicating that a polymorphism between *CYP19A1* and *ESR1* may be expressively related to POF and that the biological pathways may be involved in the regulation of folliculogenesis [24]. These theories corroborate our results that showed an increased risk of developing POF associated with the *TC/CC* genotype.

There also appears to be an increased prevalence of tubal pathology in carriers of the *TC/CC* genotype. Compelling evidence suggests that estrogen is directly related to the normal morphology and functional integrity of the Fallopian tube [27,28]. The Fallopian tube is a dynamic tissue responsive to steroids [27,61]. This theory was confirmed by an in vitro study that reports that the initial epithelial deciliation in human Fallopian tubes can be prevented by increasing estradiol levels [62]. Additionally, studies have shown that estradiol regulates tubal protein secretion in human Fallopian tubes in vivo [28,63] and in vitro [64]. As is known, estradiol production is highly upregulated during human pregnancy [65]. Estradiol is also involved in blastocyst hatching [66]. Therefore, inappropriate tubal implantation can occur if estradiol levels are changed. To infer the involvement of this polymorphism in the etiopathology of the tubal factor seems logical, considering that the synthesis of the steroid hormone is controlled by several enzymes of cytochrome P450 that are highly selective to the substrate and that the synthesis of estradiol requires cytochrome P450 aromatase, that controls the aromatization of androgens into estrogens [28,67].

A statistical association of *TC/CC* genotype with female infertility, nevertheless of associated cause, was found. This can be explained because, as we described above, the level of estrogens is highly influenced by the aromatase enzyme that is encoded by the *CYP19A1* gene. Therefore, polymorphisms of the *CYP19A1* gene will alter the activity of aromatase, and, consequently, the level of estrogens, which can lead to several diseases, including infertility.

We interestingly found that the *TC/CC* variant seems to be associated with the development of endometriosis and speculated about the possible role of this polymorphism in augmenting *CYP19A1* enzymatic activity, and thus enhancing estrogen production; the results obtained for PCOS and POF, diseases that are associated with lower estrogen levels, led us to doubt about our speculation of the role of the polymorphism on the protein activity. However, we should not forget that the human *CYP19A1* gene has an unusually large regulatory region containing 10 tissue-specific promoters that are alternatively used in different cell types. In addition to the 10 tissue-specific promoters, humans have at least eight additional promoters. The activation of the tissue-specific promoters in the different polymorphic variants may also be affected and influence the activity of the codified protein and vary estrogens levels in different tissues [68].

Regarding *GSTs* polymorphisms, our group previously confirmed a significantly increased risk of infertility associated with *GSTT1* and *GSTM1* null genotypes, alone or in association [37]. Grouping *CYP19A1* and *GSTM1* genotypes, female infertility susceptibility was altered until when *GSTM1* was present, as well as for carriers of *GSTM1* null plus *CYP19A1 C* allele. However, for *GSTM1* null genotype carriers along with simultaneous *CYP19A1 TT* genotype, this association appears not to be present. In the analysis of the combination of *CYP19A1* and *GSTT1* genotypes, independently of *GSTT1* and *CYP19A1* polymorphisms, a statistically significant association was found with the risk of developing female infertility. As far as we know, these are the first published results regarding the potential role of *CYP19A1* codon 39 polymorphism and its potential effect together with *GSTM1* and *GSTT1* in the development of female infertility. We found that homozygosity or heterozygosity for the *C* allele was significantly associated with an increased risk of infertility in the study population, and we again demonstrated the impact of *GSTM1* and *GSTT1* polymorphisms on female infertility.

Recent research is focusing on the role of oxidative stress in the pathophysiology of endometriosis, which may cause a systemic inflammatory response in the abdominal cavity [69,70]. Loss of *GSTT1* and *GSTM1* gene activity can enhance and increase ROS production, thereby promoting DNA damage and apoptosis and preventing endometrial cell proliferation and invasion [71,72], although reports also show conflicting results in different populations [14,73,74]. Our results support the idea that ROS and antioxidant imbalances are closely associated with female infertility as it affects the physiological function of the reproductive tract and affects oocyte maturation through fertilization, embryonic development, and pregnancy [33]. Male infertility is also caused by oxidative stress, which can have damaging effects on sperm structure and function [74]. Excess production of ROS by mitochondria is also associated with male infertility [34,75]. In a healthy body, ROS and antioxidants are in balance. Oxidative stress occurs when the balance is disturbed by large amounts of ROS. Cells have developed multiple antioxidant systems to reduce and deactivate ROS and repair cellular damage. The GST family plays an imperative role in the detoxification of environmentally toxic compounds and metabolites of oxidative stress, counterbalancing the production of ROS [33].

## 5. Conclusions

In conclusion, our results add important information to the identification of genetic biomarkers that contribute to the diagnosis and prognosis of infertility and to identify molecular targets that can be used for personalized treatment and prevention. This work highlights the great influence of *CYP19A1* codon 39 Trp/Arg (*T*/*C*) polymorphism (rs2236722) and *GSTM1* and *GSTT1* polymorphisms in female fertility and brings to discussion the value of assessing low penetrance polymorphisms to prevent infertility in the general population of women.

Differences in populations, along with interactions between different genes and genetic interactions with the environment, may clarify the varying results. Another limitation of this study is the likely small sample dimensions. Additional research is needed to assess the value of these polymorphisms in relation to other genes, fertility treatment outcomes, and environmental and lifestyle factors.

## Figures and Tables

**Table 1:** *CYP19A1* codon 39 Trp/Arg polymorphisms.

*CYP19A1* Codon 39 Trp/Arg Polymorphisms		
Primer FW1	5′-ATCTGTACTGTACAGCACC-3′	Primers FW1 and RV1 allow the genotyping of the *C* allele (Arg) Amplicon length: 264 bp
Primer RV1	5′-ATGTGCCCTCATAATTCCG-3′	
Primer FW2	5′-GGCCTTTTTCTCTTGGTGT-3′	Primers FW2 and RV2 allow the genotyping of the T allele (Trp) Amplicon length: 200 bp
Primer RV2	5′-CTCCAAGTCCTCATTTGCT-3′	
The total length of the amplified region (Primer FW1–Primer RV2)		Amplicon length: 427 bp

Trp/Arg, tryptophan/arginine; FW1, forward 1; RV1, reverse 1; FW2, forward 2; RV2, reverse 2.

**Table 2:** *GSTM1/GSTT1* polymorphisms.

*GSTM1* Null Polymorphism		
Primer FW	5′-GAACTCCCTGAAAAGCTAAAG-3′	Amplicon length Genotype present: 215 bp Genotype null: no amplification
Primer RV	5′-GTTGGGCTCAAATATACGGTGG-3′	
*GSTT1* null polymorphism		
Primer FW	5′-TTCCTTACTGGTCCTCACATCTC-3′	Amplicon length Genotype present: 480 bp Genotype null: no amplification
Primer RV	5′-TCACCGGATCATGGCCAGCA-3′	
*β-globin* (housekeeping gene)		
Primer FW	5′-CAACTTCATCCACGTTCACC-3′	Amplicon length: 268 bp
Primer RV	5′-GAAGAGCCAAGGACAGGTAC-3′	

FW, forward 1; RV, reverse.

**Table 3:** Clinicopathological characteristics of cases (infertile women) and controls (fertile women).

	Cases, N (%) Mean ± SD	Controls, N (%) Mean ± SD	*p*-Value
Total	201 (100.0)	161 (100.0)	NA
Age (Years)	34 ± 3.86	31 ± 4.31	
≤30 years	35 (17.4)	80 (49.7)	Reference
>30 years	166 (82.6)	81 (50.3)	*p* < 0.001
Age at Menarche (Years) *	12 ± 1.80	12 ± 1.43	
≤12 years	114 (57.6)	38 (52.1)	Reference
13–14 years	60 (30.3)	30 (41.1)	*p* = 0.107
≥15 years	24 (12.1)	5 (6.8)	*p* = 0.260
Number of Previous Pregnancies			
0:	151 (75.1)	0 (0.0)	**
1:	41(20.4)	98 (60.9)	Reference
2:	5 (2.5)	52 (32.3)	*p* < 0.001
3:	4 (2.0)	11 (6.8)	*p* = 0.541
Number of Children			
0:	180 (89.6)	0 (0.0)	**
1:	20 (10.0)	98 (60.9)	Reference
2:	1 (0.5)	52 (32.3)	*p* = 0.003
3:	0 (0.0)	11 (6.8)	**
Infertility Time (Months)	51 ± 31.9		NA
<24 months	12 (6.0)		
24–48 months	123 (61.2)	NA	
>48 months	66 (32.8)		
Smoke Habits		***	NA
Never	150 (74.6)		
Previous	25 (12.4)		
Present	26 (13.0)		
Ethnicity			NA
Caucasian	196 (97.5)	150 (93.2)	
Gipsy	3 (1.5)	9 (5.6)	
Afro-Europeans	2 (1.0)	2 (1.2)	
BMI (kg/m^2^)	24 ± 3.86	***	NA
≤25 kg/m^2^	137 (68.2)		
>25 kg/m^2^	64 (31.8)		

SD, standard deviation; BMI, body mass index; NA, not applicable. * Missing data from 3 women in cases and 88 women in controls. ** Cell counts less than 1. *** Data on smoke habits and BMI was not available for all the women included in the control group.

**Table 4:** Distribution of *CYP19A1* codon 39 polymorphisms in women with endometriosis and controls (fertile women).

	Controls, N (%)	Cases, N (%)	OR (95% CI)	*p*-Value
*TT*	96 (59.6)	12 (24.5)	1.0	
*TC/CC*	65 (40.4)	37 (75.5)	4.554 (2.209–9.386)	<0.001

OR, odds ratio; CI, confidence intervals; N, number of cases.

**Table 5:** Distribution of *CYP19A1* codon 39 polymorphisms in women with PCOS and controls (fertile women).

	Controls, N (%)	Cases, N (%)	OR (95% CI)	*p*-Value
*TT*	96 (59.6)	15 (21.7)	1.0	
*TC/CC*	65 (40.4)	54 (78.3)	5.317 (2.767–10.215)	<0.001

OR, odds ratio; CI, confidence intervals; N, number of cases.

**Table 6:** Distribution of *CYP19A1* codon 39 polymorphisms in women with POF and controls (fertile women).

	Controls, N (%)	Cases, N (%)	OR (95% CI)	*p*-Value
*TT*	96 (59.6)	14 (30.4)	1.0	
*TC/CC*	65 (40.4)	32 (69.6)	3.376 (1.672–6.815)	0.001

OR, odds ratio; CI, confidence intervals; N, number of cases.

**Table 7:** Distribution of *CYP19A1* codon 39 polymorphisms in women with tubal pathologies and controls (fertile women).

	Controls, N (%)	Cases, N (%)	OR (95% CI)	*p*-Value
*TT*	96 (59.6)	16 (31.4)	1.0	
*TC/CC*	65 (40.4)	35 (68.6)	3.231 (1.653–6.314)	0.001

OR, odds ratio; CI, confidence intervals; N, number of cases.

**Table 8:** Distribution of *CYP19A1* codon 39 polymorphisms in cases (infertile women) and controls (fertile women).

	Controls, N (%)	Cases, N (%)	OR (95% CI)	*p*-Value
*TT*	96 (59.6)	52 (31.4)	1.0	
*TC/CC*	65 (40.4)	149 (68.6)	4.232 (2.710–6.609)	<0.001

OR, odds ratio; CI, confidence intervals; N, number of cases.

**Table 9:** Association between *CYP19A1* and *GSTM1* genotype combinations and female infertility.

CYP19A1	GSTM1	Controls, n (%)	Cases, n (%)	OR (95% CI)	*p*-Value
TT	+	49 (30.4)	24 (11.9)	1.0	
TC/CC	+	40 (24.9)	63 (31.4)	3.216 (1.715–6.031)	<0.001
TT	-	47 (29.2)	28 (13.9)	1.216 (0.618–2.392)	0.346
TC/CC	-	25 (15.5)	86 (42.8)	7.023 (3.627–13.601)	<0.001

OR, odds ratio; CI, confidence intervals; N, number of cases.

**Table 10:** Association between *CYP19A1* and *GSTT1* genotype combinations and female infertility.

CYP19A1	GSTT1	Controls, N (%)	Cases, N (%)	OR (95% CI)	*p*-Value
TT	+	84 (52.2)	15 (7.5)	1.0	
TC/CC	+	49 (30.4)	80 (39.8)	9.143 (4.752–17.591)	<0.001
TT	-	12 (7.5)	37 (18.4)	17.267 (7.366–40.476)	<0.001
TC/CC	-	16 (9.9)	69 (34.3)	24.150 (11.148–52.317)	<0.001

OR, odds ratio; CI, confidence intervals; N, number of cases.

**Table 11:** Association between *CYP19A1*, *GSTM1*, and *GSTT1* genotype combinations and female infertility.

CYP19A1	GSTM1	GSTT1	Controls, N (%)	Cases, N (%)	OR (95% CI)	*p*-Value
TT	+	+	39 (24.2)	7 (3.5)	1.0	
TC/CC	+	+	29 (18.0)	37 (18.4)	7.108 (2.777–18.197)	<0.001
TT	+	-	10 (6.2)	17 (8.5)	9.471 (3.086–29.067)	<0.001
TC/CC	+	-	11 (6.9)	26 (12.9)	13.169 (4.518–38.380)	<0.001
TT	-	+	43 (26.7)	8 (4.0)	1.037 (0.344–3.124)	0.587
TC/CC	-	+	20 (12.4)	43 (21.4)	11.979 (4.570–31.400)	<0.001
TT	-	-	4 (2.5)	20 (9.9)	27.857 (7.283–106.551)	<0.001
TC/CC	-	-	5 (3.1)	43 (21.4)	47.914 (14.051–163.393)	<0.001

OR, odds ratio; CI, confidence intervals; N, number of cases.

## Data Availability

The data are contained within this article.
